# Viscosity effects of polyvinyl siloxane impression materials on the accuracy of the stone die produced

**Published:** 2018-04-12

**Authors:** Warren Ho, Liang Lin Seow, Ammar Musawi

**Affiliations:** ^1^Drs. Wong & Partner Dental Surgeons, KualaLumpur, Malaysia; ^2^International Medical University, Kuala Lumpur, Malaysia; ^3^Missouri School of Dentistry and Oral Health, A. T. Still University, Kirksville, Missouri, United States

**Keywords:** polyvinyl siloxane, final impression, viscosity

## Abstract

**Background:**

The purpose of the present study was to investigate the effect of different viscosities of polyvinyl siloxane (PVS) impression materials on the accuracy of the stone die produced.

**Methods:**

A three-unit bridge master model was fabricated using cold-cure acrylic resin. Four combinations of different viscosities of PVS impression materials - regular body (monophase) alone, light body with regular body, light body with heavy body, and light body with putty - were used to make an impression of the master model. Ten impressions from each group were taken and Type IV gypsum stone was used to generate the dies. The dies were measured at the inter-abutment distance, occlusogingival length, and shoulder width with a measuring microscope and were compared with the master model using one-way analysis of variance and Tukey (honest significant difference) test.

**Results:**

Differences were found for inter-abutment distance between the master model and the light body with regular body and light body with putty dies (both *P* < 0.02). A difference was found for shoulder width between the master model and the regular body alone die (P = 0.01). No differences were found for occlusogingival distance (all *P* > 0.08).

**Conclusion:**

Results suggested inter-abutment distance was most accurate when using a PVS light body combination. Occlusogingival length was accurate using any of the studied PVS combinations, and shoulder width was more accurate when using the regular body PVS.

**Relevance for patients:**

These results should be considered when choosing the viscosity of the PVS to use for producing impressions of high accuracy and fabricating a well-fitting fixed prosthesis.

## 1. Introduction

The ability to record the tooth preparation accurately during the impression stage is critical for producing well-fitting crowns and bridges. Impression materials that exhibit good dimensional stability and accuracy are necessary to record the fine details of hard and l tissues and obtain biologically, mechanically, functionally, and esthetically acceptable restorations[1-3].

Synthetic elastomeric impression materials were first introduced in the late 1950s and became popular because they improved on the existing hydrocolloid impression materials in two aspects, namely; dimensional stability and inadequate tear resistance associated with the hydrocolloid material used at that time[4]. There are currently four types of elastomeric impression materials used for crown and bridge work in dentistry: polysulfide, condensation polyvinyl siloxane, addition polyvinyl siloxane, and polyether.

At present, addition polyvinyl siloxane is widely used because of its high accuracy, good dimensional stability, good elastic properties, high tear strength, excellent recovery from deformation on removal, and short working and setting time[5-7]. Further, this impression material does not have polymerization shrinkage like that of condensation polyvinyl siloxane, so that it produces a highly stable impression because no by-products are released during polymerization[8]. Addition polyvinyl siloxane has superior results for accuracy and dimensional stability compared with condensation polyvinyl siloxane, and research indicates that addition polyvinyl siloxane remains unchanged over time, allowing impressions to be poured days after they were recorded [5,9]. Polysulfide is a relatively unpopular impression material because of its long setting time. It is also messy to handle and has an unpleasant odor. Polyether impression materials have adequate tear resistance and good elastic properties, but they have high elastic modulus and are relatively rigid when set. As such, considerable force may be required to remove the impression from the mouth or the stone cast[4].

Addition polyvinyl siloxane impression materials have four types of viscosity to suit different needs. The light body has the lowest viscosity and is placed on hard and soft tissues to record accurate surface details of tooth preparations. However, the light body has inadequate dimensional stability to maintain its form during production of the working cast. The medium body is commonly used as a monophase material or single-viscosity technique during crown and bridge work or for dentures. The heavy body has a higher viscosity and is generally placed in the impression tray to support the light body material for crown and bridge impressions. Putty has high filler loading and exhibits significant polymerization shrinkage. It is often combined with a low-viscosity silicone during the impression procedure, known as the putty-wash technique, and is commonly used for dental impressions[9].

Two techniques—one-stage or two-stage procedures—can be used when taking impressions with light body and putty materials. For one-stage procedures, the wash material is syringed on the prepared tooth, and then the unset putty is seated over the light body material. For two-stage procedures, the initial putty impression is made and allowed to set, and then it is subsequently relined with wash materials [10,11].

Hung et al.[12] reported a single-step technique that had greater accuracy than a double-mix technique, mainly because double-mix techniques require dimensional alterations, extra chair side time, and extra material. In another study, putty-wash and monophase impression techniques were shown to be equally accurate at recording tooth preparation[13]. In most clinical situations, putty and heavy body impression materials have high consistency, but they tend to displace the light body material, which may affect the accuracy of impression[8]. The viscosity of the impression materials used increases with the proportion of fillers present. For instance, light body has low filler content and responds to high shear stresses; therefore, it gets displaced, this outcome is known as the shear thinning effect.

The objective of the present study was to investigate the effect of different viscosities of polyvinyl siloxane impression materials on the accuracy of the stone model produced with a one-stage, putty-wash impression technique. The outcomes of this research may serve as a guide to clinicians for choosing the viscosity of the polyvinyl siloxane material that will produce impressions of high accuracy and result in the fabrication of a well-fitting fixed prosthesis.

2. Materials and Methods

**Figure 1.**
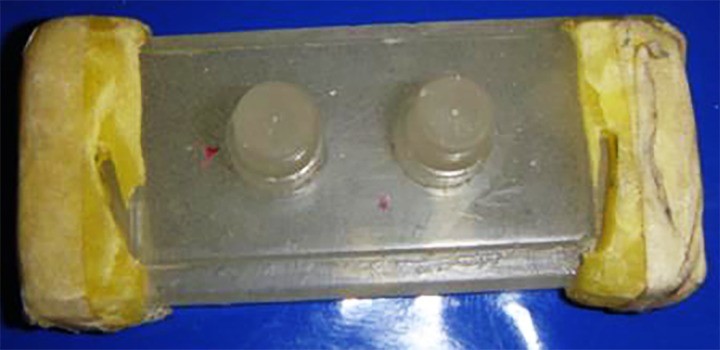
Three-unit fixed partial denture master model.

A simulated three-unit bridge master model was fabricated using acrylic resin (Figure 1). A dimple was created on the occlusal surface of both abutments as a reference point for measurement of inter-abutment distance (Figure 2A). Grooves were created on the axial surface and shoulder margin of the abutment for measurement of the occlusogingival length and shoulder width, respectively (Figure 2B).

**Figure 2A.**
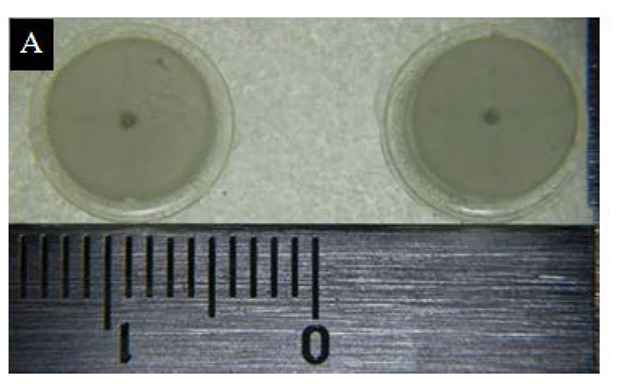
Perforated sectional tray with grooves on the base of the master model.

**Figure 2B.**
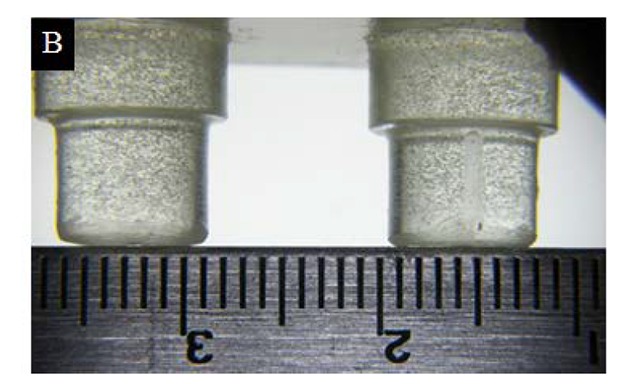
Groove on the abutment surface.

**Figure 3.**
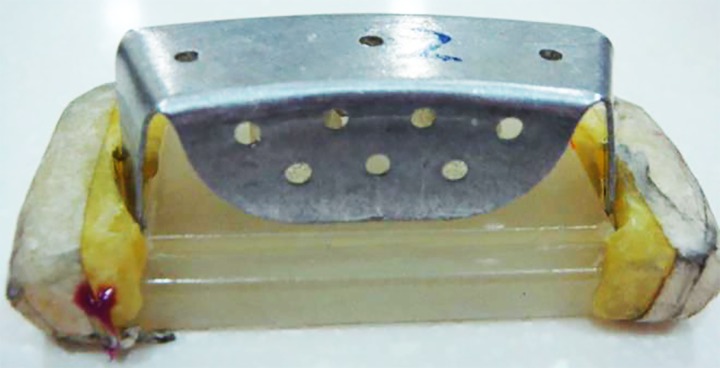
Perforated sectional tray with grooves on the base of the master model.

Measurements were made and compared between the master model and the produced die models for the inter-abutment distance, occlusogingival length, and shoulder width. A perforated sectional tray was used to make the impression of the master model. In addition, two grooves were created on the base of acrylic resin model for the tray to be placed accurately on the master model during the impression taking procedure and to standardize the path of insertion and removal (Figure 3).

Four combinations of different viscosities of the polyvinyl siloxane impression material were used to take the impression of the master model: regular body (monophase) alone, light body with regular body, light body with heavy body, and light body with putty (Aquasil, DENTSPLY Caulk, Milford, DE, USA). The one-stage impression technique was used for taking the impression of the acrylic resin master model. The wash material was syringed on the master model, and then the unset putty was seated over the light body material. Ten impressions were taken for each of the four viscosities for a total of 40 impressions.

Impression materials were delivered using a syringe gun. The amount of monophase, heavy body, and putty impression materials in the tray were weighed with a digital weighing device to ensure a standardized amount of material was used. After dispensing, the material was allowed to set; we doubled the recommended setting time to ensure a proper set of the material. Because this procedure was carried out at the bench top and not in a clinical setting, we were able to double the time. All impressions were stored at a room temperature (25 °C) for 1 hour before pouring the stone to make the die models. Impressions that had voids were discarded and a new impression was made.

Type IV gypsum stone was used to generate the die models. The stone powder was mixed by hand at a ratio of 10 g of stone powder to 6 mL of water, following the manufacturer's instructions. The mixture was poured into the impression incrementally using a vibrator to prevent air bubble entrapment. The die was allowed to set and was removed from the impression 1 hour after pouring. Die models were left at room temperature to dry.

Photographs of the master model and dies were taken with a Nikon Model D60 camera attached to a microscope. Photographs were transferred to a computer, and measurements (in mm) of inter-abutment distance, occlusogingival length, and shoulder width were made using measuring software (Nis-Element, Nikon, Melville, NY, USA). Each measurement was performed twice, and the mean value recorded. The data were inserted into SPSS statistical software (IBM, Armonk, NY, USA, Version 8.0) and analyzed using one-way analysis of variance and Tukey (honest significant difference) test to compare the means. A P-value of < 0.05 was considered statistically significant.

## 3. Results

Table 1contains the mean (SD) for the inter-abutment distance, occlusogingival length, and the shoulder width for the 4 dies and the master model. Significant difference was found between the 4 dies and the master model only with respect to inter-abutment distance (P = 0.02) and the shoulder width (P = 0.02). No difference was found between 4 dies and master model for occlusogingival length (P = 0.08). P-values for the multiple comparisons are listed in Table 2. For inter-abutment distance, differences were found between the master model and the light body with regular body and light body with putty dies (both *P* < 0.02). In terms of shoulder width, a difference was found only between the master model and the regular body (monophase) die *(P* = 0.01).

**Table 1.**
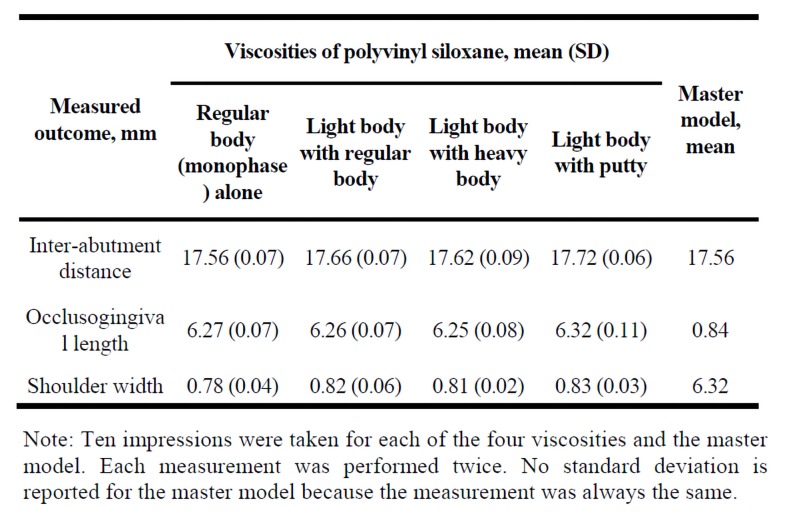
Inter-abutment distance, occlusogingival length, and shoulder width for the master model and four stone dies with different viscosities of polyvinyl siloxane.

note Ten impressions were taken for each of the four viscosities and the master model. Each measurement was performed twice. No standard deviation is reported for the master model because the measurement was always the same.

For inter-abutment distance, the mean (SD) for the dies ranged from 17.56-17.72 mm (0.06-0.09 mm); the master model measured 17.56 mm (Table 1). For occlusogingival length, the mean (SD) for the dies ranged from 6.25-6.32 mm (0.07-0.11 mm); the master model was 6.32 mm. For shoulder width, the mean (SD) for the dies ranged from 0.78-0.83 mm (0.02-0.06 mm); the master model was 0.84 mm.

Differences were found between the master model and the dies (Table 2). For inter-abutment distance, differences were found between the master model and the light body with regular body and light body with putty dies (both PO.02). No differences were found for occlusogingival distance (all *P* > 0.08). For shoulder width, a difference was found between the master model and the regular body (monophase) alone die *(P* = 0.01).

**Table 2.**
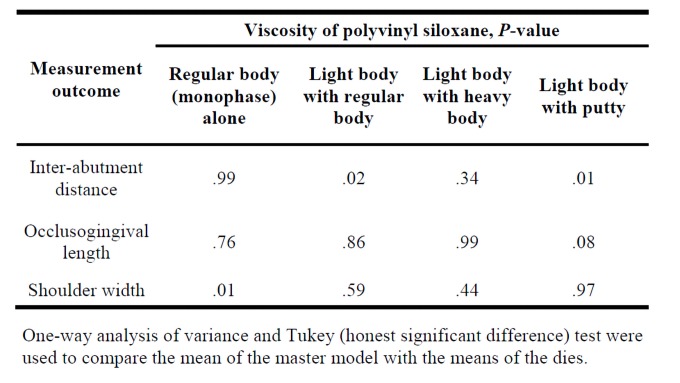
Comparisons for inter-abutment distance, occlusogingival length, and shoulder width between the master model and the four stone dies with different viscosity of polyvinyl siloxane.

One-way analysis of variance and Tukey (honest significant difference) test were used to compare the mean of the master model with the means of the dies.

## 4. Discussion

The aim of this study was to investigate the accuracy of the stone model poured from different PVS impression material viscosities and techniques (combinations). The techniques used were one stage impression and the putty-wash impression techniques. In the present study, inter-abutment distance was most accurate when using a polyvinyl siloxane material having a viscosity with a light body combination. Occlusogingival length was accurate using any of the studied viscosity combinations, and shoulder width was more accurate when using a polyvinyl siloxane material with the regular body (monophase) viscosity.

The inter-abutment distance in the present study increased for the die models produced from light body with regular body and light body with putty compared with the master model. Gordon et al.[14] reported that inter-abutment distances of stone die models were greater when using polysulfide, polyether, and with the addition PVS impression materials. Wassell and Ibbetson[15] reported that heavy body and wash impressions were more accurate than putty-wash impressions. In another study, Federick and Caputo[16] showed that the putty-wash technique was significantly less accurate than polyether (heavy and light body) or reversible hydrocolloid impressions. Clinically, this reduced accuracy may result in the fabrication of bridges that are slightly too long mesiodistally, so that the fitting would be tight and inadequate space would be left for luting cement. For an implant-supported bridge, there may not be passive fitting and increased stress and strains in the implants will result. These observed increases in the inter-abutment distance are likely the result of the linear setting expansion of the stone die material. This linear contraction is well matched to the setting expansion of modern Type III and Type IV die stones and results in a slightly larger replication of the preparation[17]. Another reason for this may be because the putty impression material shrinks toward the perforated trays as it sets so that the perforations engage the putty material. In general, the small differences in dimensional accuracy among the polyvinyl siloxane materials can be attributed to the variability in the composition of each brand name, mainly in the matrix-filler ratio, which can provide the material with different levels of polymerization shrinkage and elastic recovery [18,19].

For occlusogingival length, all the tested viscosities seemed to have comparable accuracy and dimensional stability. There were no statistically significant differences between master model and the dies. Therefore, for a single-unit crown, all the studied combinations of viscosity would be able to produce an accurate stone die from the impression for occlusogingival length.

In the present study, the shoulder width of dies produced from light body material combined with more viscous polyvinyl siloxane materials were not statistically different compared with the master model; the regular body (monophase) alone material was significantly different. This finding is results from the ability of the light body material to accurately record fine details of the shoulder margin. We observed that the more viscous polyvinyl siloxane materials displaced the light body apically when making impressions, so that the light body materials were able to register the shoulder margin more accurately. The international standard for dental elastomeric impression materials indicates that a Type III (light body) impression material must reproduce a line 0.020 mm in width. Ciesco et al.[19] showed that very low viscosity materials reproduced lines 1-2 Lim wide, which supports the results of the present study.

In the present study, specific methods and techniques were used to reduce possible errors. Impressions were taken in dry conditions in the present study. Research has shown that there is no significant adverse effect on the dimensional accuracy of silicone impressions in dry, moist, or wet conditions, but the best surface detail results were obtained under dry conditions[20], And this could be a limitation of this study. The cartridge-mix technique was used in the present study. In one study, no significant difference in dimensional changes was found when hand- and cartridge-mix techniques of polyvinyl siloxane were compared[21]. We used the one-stage impression technique in the present study. Comparisons of the monophase and two-stage putty-wash techniques in relation to the one-stage putty-wash technique showed a significantly better correspondence of the three-dimensional reproduction of the prepared teeth by one-stage techniques[22]. In the present study, the setting time for each impression was doubled to ensure a proper set of the material because the procedure was conducted at room temperature. A digital weighing machine was used to standardize the amount of materials used to reduce confounding variables. Finally, a dimple was created on the abutment surface instead of cross grooves because measurements were more accurate on pinpoint references.

## 5. Conclusion

The light body with putty combination produced a die with an increased inter-abutment distance. This result may have implications for clinicians when fabricating bridges. The occlusogingival length was accurate using all the combinations of different viscosites of polyvinyl siloxane impression materials. The light body supported by more viscous polyvinyl siloxane impression material was able to record the shoulder width more accurately and may produce crowns with an accurate marginal fit.
